# Glaucoma Surgery Calculator: Limited Additive Effect of Phacoemulsification on Intraocular Pressure in Ab Interno Trabeculectomy

**DOI:** 10.1371/journal.pone.0153585

**Published:** 2016-04-14

**Authors:** Ashley E. Neiweem, Igor I. Bussel, Joel S. Schuman, Eric N. Brown, Nils A. Loewen

**Affiliations:** 1 Rosalind Franklin University of Medicine and Science, Chicago Medical School, Chicago, United States of America; 2 Department of Ophthalmology, School of Medicine, University of Pittsburgh, Pittsburgh, United States of America; 3 Department of Ophthalmology, School of Medicine, Vanderbilt University, Nashville, Tennessee, United States of America; Duke University, UNITED STATES

## Abstract

**Purpose:**

To compare intraocular pressure (IOP) reduction and to develop a predictive surgery calculator based on the results between trabectome-mediated ab interno trabeculectomy in pseudophakic patients versus phacoemulsification combined with trabectome-mediated ab interno trabeculectomy in phakic patients.

**Methods:**

This observational surgical cohort study analyzed pseudophakic patients who received trabectome-mediated ab interno trabeculectomy (AIT) or phacoemulsification combined with AIT (phaco-AIT). Follow up for less than 12 months or neovascular glaucoma led to exclusion. Missing data was imputed by generating 5 similar but non-identical datasets. Groups were matched using Coarsened Exact Matching based on age, gender, type of glaucoma, race, preoperative number of glaucoma medications and baseline intraocular pressure (IOP). Linear regression was used to examine the outcome measures consisting of IOP and medications.

**Results:**

Of 949 cases, 587 were included consisting of 235 AIT and 352 phaco-AIT. Baseline IOP between groups was statistically significant (p≤0.01) in linear regression models and was minimized after Coarsened Exact Matching. An increment of 1 mmHg in baseline IOP was associated with a 0.73±0.03 mmHg IOP reduction. Phaco-AIT had an IOP reduction that was only 0.73±0.32 mmHg greater than that of AIT. The resulting calculator to determine IOP reduction consisted of the formula -13.54+0.73 × (phacoemulsification yes:1, no:0) + 0.73 × (baseline IOP) + 0.59 × (secondary open angle glaucoma yes:1, no:0) + 0.03 × (age) + 0.09 × (medications).

**Conclusions:**

This predictive calculator for minimally invasive glaucoma surgery can assist clinical decision making. Only a small additional IOP reduction was observed when phacoemulsification was added to AIT. Patients with a higher baseline IOP had a greater IOP reduction.

## Introduction

Cataract surgery is often associated with a moderate intraocular pressure (IOP) reduction of 1.5–3 mmHg in patient with ocular hypertension or glaucoma [1–3]. Minimally invasive glaucoma surgery (MIGS) allows to combine IOP lowering with vision improvement from cataract surgery in an age group often affected by both. Such a practice pattern has become more common because it is standardized, safe [4] and also cost effective [5]. The first randomized controlled trials comparing phacoemulsification alone to phacoemulsification combined with implantation of trabecular bypass microstents, a form of MIGS, (iStent, Glaukos, Laguna Hills, CA), showed a relatively small additional effect of these implants on IOP reduction [6]. Ab interno trabeculectomy with the trabectome (Neomedix Corp; Tustin, CA), another MIGS modality, lowers IOP by plasma-mediated ionization and ablation of trabecular meshwork (TM) of up to 180° thereby increasing aqueous outflow in eyes with an intact downstream drainage system [4]. Both ab interno trabeculectomy (AIT) and phacoemulsification combined with ab interno trabeculectomy (phaco-AIT) can be used in patients with different angle opening [7] and surgical status [8,9]. The purpose of the comparison in this study was to assess reduction of IOP after AIT performed in pseudophakic patients versus phaco-AIT in phakic patients using a *Coarsened Exact Matched* cohort. Based on results with microstents [10], we hypothesized that in this matched comparison the benefit of adding phacoemulsification to AIT would be associated with a greater reduction in IOP and medications during 12 month follow-up. The resulting calculator can help clinicians to predict the IOP reduction.

## Methods

### Participants

Data for this study were collected with approval by the Institutional Review Board of the University of Pittsburgh, in accordance with the Declaration of Helsinki and the Health Insurance Portability and Accountability Act. No informed consent was necessary for this retrospective, observational cohort study. Patient records were anonymized and de-identified prior to analysis. Subjects were divided into pseudophakic patients who received AIT and phakic patients who received phaco-AIT. Outcomes were determined for all patients with a diagnosis of glaucoma with or without a visually significant cataract, who had 12 months of follow-up. The specific target IOP was set on a case-by-case basis by the individual treating physician and was the maximum IOP estimated to prevent further nerve damage. Patients who were followed for less than 12 months or diagnosed with neovascular glaucoma were excluded. Indications for AIT consisted of worsening glaucoma on maximally tolerated topical therapy while indications for phaco-AIT were the same or stable glaucoma with desire to reduce medications plus a visually significant cataract with visual brightness acuity testing equal or worse than least 0.4 logMAR (20/50 Snellen). The postoperative medications consisted of 1% pilocarpine four times per day for 1 month, then three times per day for 1 month, 1% prednisolone acetate four times per day for 1 week to be tapered by one drop each week, and a third or fourth generation fluoroquinolone four times per day for 1 week. Glaucoma medications could be continued as deemed necessary to achieve target pressures. Visual field status of all patients was categorized as early, moderate, or advanced by individual glaucoma specialists based on the most recent Humphrey visual field exams (Zeiss, Jena, Germany). All patients had a comprehensive slit lamp, gonioscopy and dilated ophthalmoscopy exam prior to surgery.

### Statistics

Demographics were compared by Mann-Whitney U test and chi-squared test for continuous and categorical variables, respectively. To avoid eliminating data with missing values multiple imputation was used. Missing values of the incomplete dataset were imputed *m*>1 times, thus creating *m* completed datasets. Second, each of the *m* completed datasets were independently analyzed. Finally, the results from each of the *m* analysis were pooled into a final result. Missing data such as age, gender and race were imputed by generating 5 similar but non-identical datasets. Groups were then matched by utilizing *Coarsened Exact Matching* [11–13] based on age, gender, type of glaucoma, race, preoperative number of glaucoma medications and baseline IOP. Univariate linear regression was performed first and those variables that were statistically significant were included in the final multivariate regression model. A p-value of less than 0.05 was considered statistically significant. Continuous variables were expressed as mean±SD. All analyses were performed using R [14].

## Results

### Baseline Demographics

**Fig 1:**
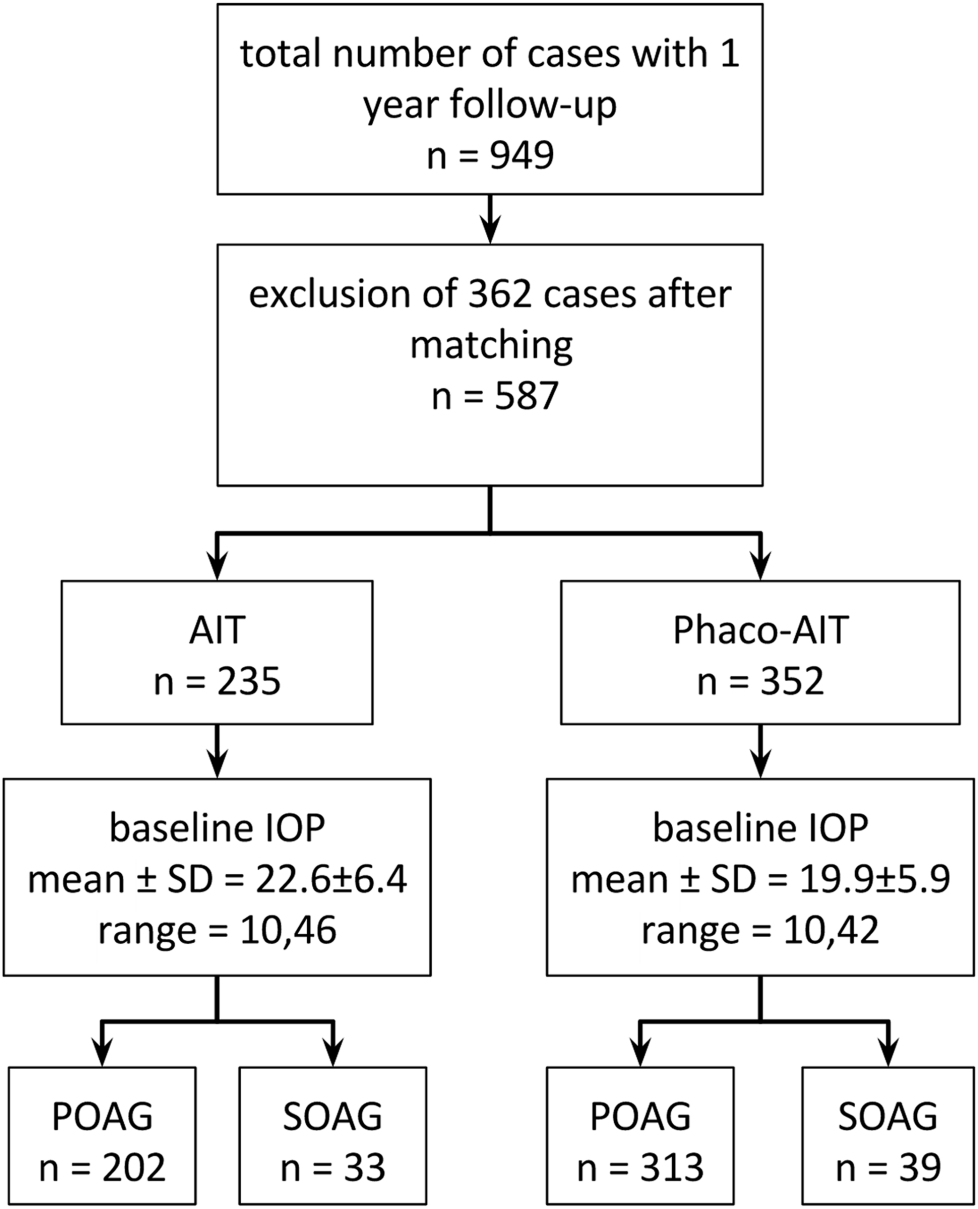
Trabectome Surgeries. Cases analyzed after accounting for exclusion criteria (AIT, ab interno trabeculectomy; phaco-AIT, AIT combined with phacoemulsification; IOP, intraocular pressure; POAG, primary open angle glaucoma; SOAG, secondary open angle glaucoma).

After applying exclusion criteria and matching, a total of 587 patients were included in the study consisting of 235 AIT and 352 phaco-AIT (Fig 1). Baseline demographics are shown in Table 1.

**Table 1:** Raw data demographics of study population. Demographics for AIT-only and phaco-AIT show significant difference (p < 0.05) for age, gender, baseline IOP, and baseline number of medications in unmatched data.

	AIT (n = 368)	Phaco-AIT (n = 581)	p-value
**Age**			<0.01
Mean±SD	75±10	74±9	
Range	(50, 96)	(51, 94)	
**Gender**			0.01
Male	130 (35%)	248 (43%)	
Female	223 (61%)	323 (56%)	
**Types of Glaucoma**			0.19
ACG	2 (1%)	11 (2%)	
POAG	289 (78%)	442 (76%)	
SOAG	77 (21%)	128 (22%)	
**Race**			0.34
African Americans	19 (5%)	29 (5%)	
Asians	92 (25%)	166 (29%)	
Caucasians	216 (59%)	303 (52%)	
Others	10 (3%)	20 (3%)	
**Baseline IOP**			<0.01
Mean±SD	24.1±7.1	20.6±6.6	
Range	(10, 51)	(10, 59)	
**Baseline Number of Glaucoma Medications**			<0.01
Mean±SD	2.9±1.1	2.4±1.0	
Range	(1, 6)	(1, 5)	

ACG (angle closure glaucoma); POAG (primary open angle glaucoma); SOAG (secondary open angle glaucoma).

ACG (angle closure glaucoma); POAG (primary open angle glaucoma); SOAG (secondary open angle glaucoma).

Primary open angle glaucoma (POAG) comprised 86% and 89% of AIT and phaco-AIT, respectively. Secondary open angle glaucoma (SOAG) included 14% and 11% of AIT and phaco-AIT, respectively. From the matched subjects, 75% in AIT and also in phaco-AIT were Caucasian, followed by Asian, African American, and others. These values were not statistically significant. Additionally, age, gender, baseline number of glaucoma medications, and baseline IOP were found to be statistically different (p<0.01) between groups (Table 1). Following *Coarsened Exact Matching*, these preoperative differences between treatment groups were minimized (Table 2).

**Table 2:** Matched data demographics of study population. Matched data is shown for both AIT-only and phaco-AIT group demographics.

	AIT (n = 235)	phaco-AIT (n = 352)	p-value
**Age**			0.04
Mean±SD	76±9	75±8	
Range	(51,96)	(56,94)	
**Gender**			0.15
Female	156 (66%)	212 (60%)	
Male	79 (34%)	140 (40%)	
**Types of Glaucoma**			0.34
ACG	0 (0%)	0 (0%)	
POAG	202 (86%)	313 (89%)	
SOAG	33 (14%)	39 (11%)	
**Race**			0.77
African Americans	7 (3%)	7 (2%)	
Asians	48 (20%)	78 (22%)	
Caucasians	176 (75%)	263 (75%)	
Others	4 (2%)	4 (1%)	
**Baseline IOP**			<0.01
Mean±SD	22.6±6.4	19.9±5.9	
Range	(10, 46)	(10, 42)	
**Baseline Number of Glaucoma Medications**			<0.01
Mean±SD	2.8±1.1	2.4±1.1	
Range	(1,6)	(1,5)	

### Multiple Imputation and Coarsened Exact Matching

Missing data in each category are recorded. Data missing from baseline number of medications, type of glaucoma, and IOP were 0% for both groups. Conversely, age, gender, and race had missing data among both groups. Six percent of AIT had an unknown age, 4% were without defined gender and 8% without defined race. Two percent of phaco-AIT had an unknown age, 2% were without defined gender, and 11% without race.

### Linear Regression Models

Linear regression of the multiple imputed, matched data was used to identify the influence of the parameters above on the IOP lowering effect of surgery. Univariate linear regression was performed first (Table 3) using the variables phacoemulsification, baseline IOP, SOAG, age, number of medications at baseline, race and gender (male).

**Table 3:** Univariate linear regression of patient parameters of study. A p-value of <0.05 is considered statistically significant.

	Coefficient	Standard Error	p-value
Phaco	-1.35	0.50	<0.01
Baseline IOP	0.74	0.03	<0.01
SOAG	4.17	0.74	<0.01
Age	0.10	0.03	<0.01
Baseline # of medications	0.56	0.22	<0.01
Race			
Asian	1.10	2.30	0.64
Caucasian	1.94	2.19	0.39
Other	4.84	3.47	0.18
Male	-0.75	0.50	0.14

Phaco (phacoemulsification); IOP (intraocular pressure); SOAG (secondary open angle glaucoma).

Of these variables, phacoemulsification, baseline IOP, SOAG, age and number of medications at baseline were found to be statistically significant and included in the final multivariate regression model (Table 4). Only baseline IOP and phacoemulsification were statistically significant in both models (p = 0.02 and p<0.01, respectively).

**Table 4:** Multivariate linear regression of patient parameters that were found to be statistically significant (p < 0.05) in univariate linear regression.

	Coefficient	Standard Error	p-value
Intercept	-13.54	1.67	<0.01
Phaco	0.73	0.32	0.02
Baseline IOP	0.73	0.03	<0.01
SOAG	0.59	0.50	0.24
Age	0.03	0.02	0.10
Baseline # of Medications	0.09	0.14	0.55

Phaco (phacoemulsification); IOP (intraocular pressure); SOAG (secondary open angle glaucoma).

**Fig 2:**
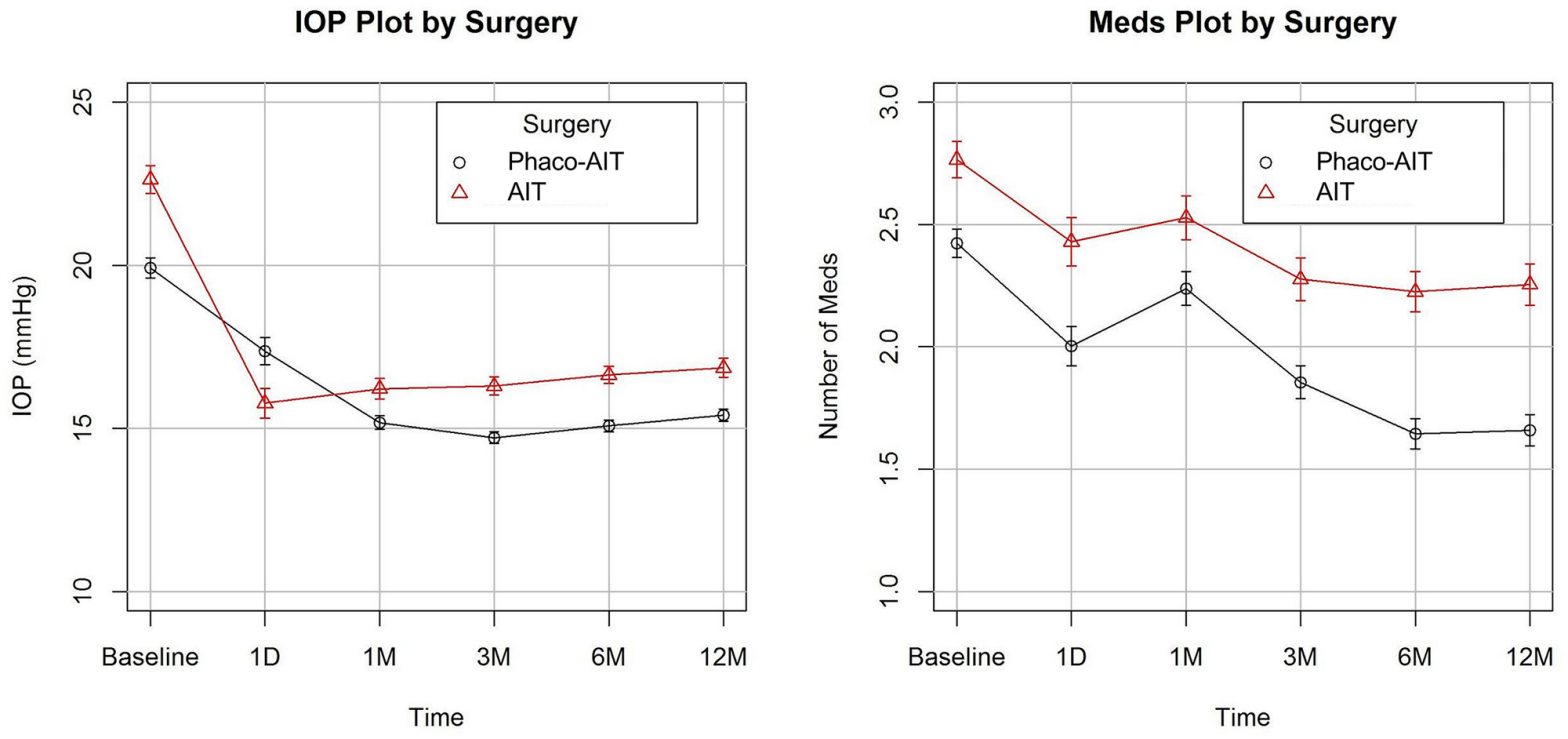
IOP and medication plots. Preoperative and postoperative intraocular pressure (IOP; left) and number of glaucoma medications (Meds; right) over the 12 month follow-up for both groups. Represented as mean ± standard error. Statistically significantly different at all time points for both plots (p<0.05).

Each increment of 1 mmHg in baseline IOP was associated with an IOP reduction of 0.73±0.03 mmHg (p<0.01). After adjusting for baseline IOP, age, baseline number of glaucoma medications, and type of glaucoma, phacoemulsification conferred an additional IOP reduction of 0.73±0.32 mmHg IOP. Preoperative IOP was 22.6±6.4 mmHg in AIT and 19.9±5.8 mmHg in phaco-AIT with 2.8±1.1 medications in AIT and 2.4±1.1 in phaco-AIT. At one year, IOP in AIT was reduced to 16.9±4.5 mmHg (mean±SD) and in phaco-AIT to 15.4±3.6 mmHg (p<0.01), while medications in AIT declined to 2.3±1.3 and in phaco-AIT to 1.7±1.2 (p<0.01). Postoperative AIT and phaco-AIT were significantly different at all time points for both IOP and medications due to the large sample sizes with a narrow confidence interval and small standard error (Fig 2).

### Glaucoma Surgery IOP Reduction Calculator

The calculator predicting the IOP reduction had the formula: *-13*.*54 + 0*.*73 × (phaco; yes*:*1*, *no*:*0) + 0*.*73 × (baseline IOP) + 0*.*59 × (SOAG; yes*:*1*, *no*:*0) + 0*.*03 × (age) + 0*.*09 × (medications)*.

For example, a 75-year-old pseudophakic patient with POAG with a baseline IOP of 21 and 2 different medications receiving AIT alone would be expressed as: 13.54+0.73(0)+0.73×(21)+0.59(0)+0.03×(75)+0.09×(2) = 4.22 mmHg reduction in IOP, with a resulting postoperative IOP of 16.78 mmHg.

Conversely, a 75-year-old phakic patient with POAG with the same baseline IOP and medications receiving phaco-AIT in a combined approach would have an IOP reduction of -13.54+0.73(1)+0.73×(21)+0.59(0)+0.03(75)+0.09(2) = 4.95 mmHg, thus a postoperative IOP of 16.05 mmHg.

**Fig 3:**
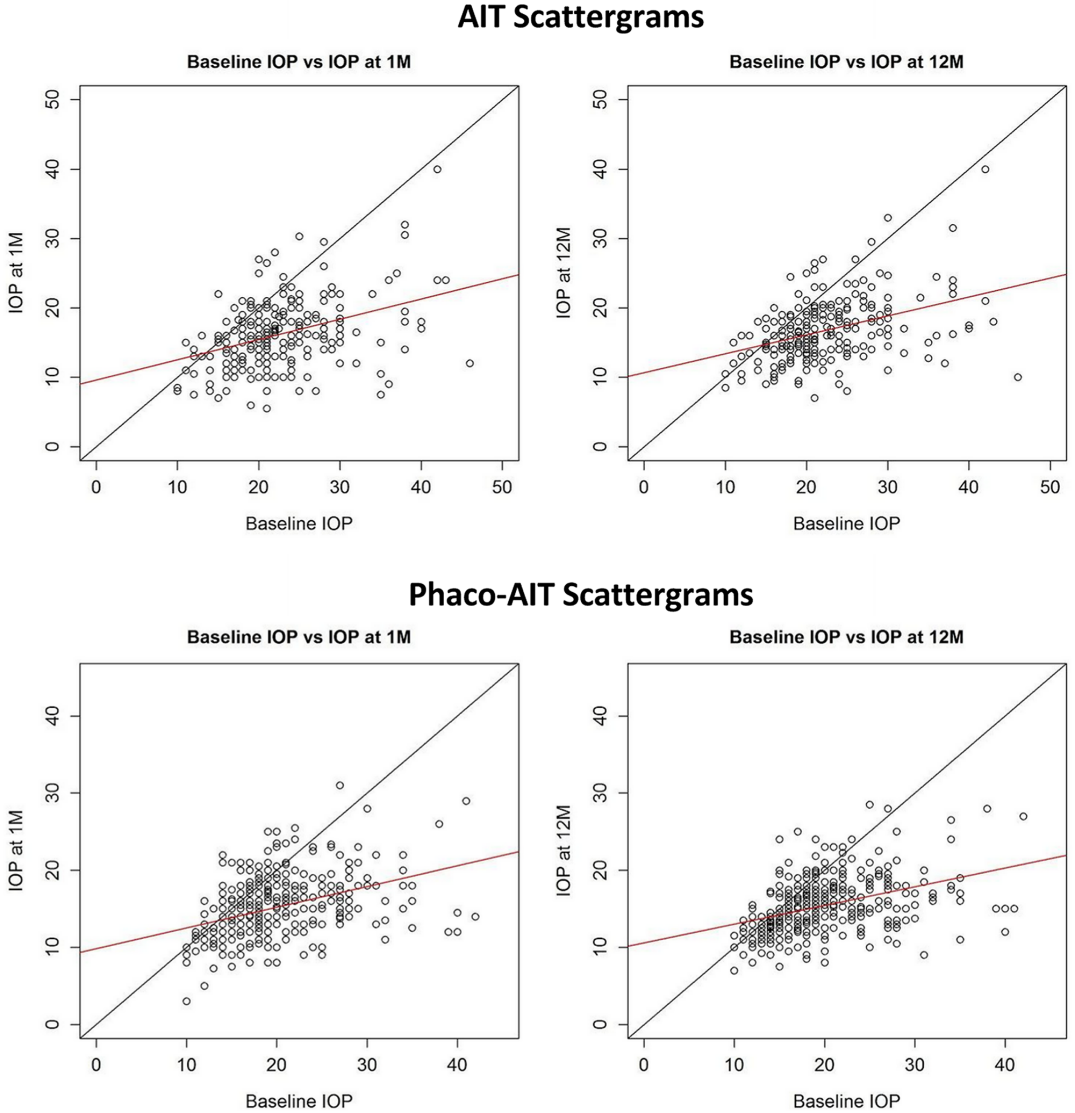
Phaco-AIT and AIT Scattergrams. Scattergrams of AIT and phaco-AIT after 1 month (left) and 12 months (right). Baseline IOP plotted against IOP at 1 month and 12 months with x = y line. Red line represents linear fit.

The linear relationship of pre- and postoperative IOP can be seen in the scattergrams that show every single data point (Fig 3).

## Discussion

We created a glaucoma surgery calculator to determine the postoperative IOP based on the preoperative IOP, type of open angle glaucoma, age, medications, and type of surgery. This first calculator for minimally invasive glaucoma surgery (MIGS) can help clinicians to estimate outcomes and anticipate the need for postoperative glaucoma medications. Using *Coarsened Exact Matching*, we found only a small additional contribution of phacoemulsification to the considerable IOP reduction from AIT. The impact of baseline IOP on total pressure reduction was substantial, adding 0.73 mmHg IOP reduction per 1 mmHg higher baseline IOP. Both groups achieved a significant decline in medications.

Compared to the limited IOP effect of only 0.73±0.32 mmHg by phacoemulsification in our study, a more significant IOP reduction can sometimes be seen after phacoemulsification [1–3]. It has been hypothesized to be caused by TM and Schlemm's canal distension that increase the outflow facility [15,16], activation of a TM stress response pathway from ultrasound and fluids [17], a trabeculoplasty-like effect [18,19] or resolution of relative pupillary block [15]. Our results are consistent with the concept that this may be mediated by the remaining temporal TM [16,20]. Different from the open angle glaucoma patients examined in this study, angle closure glaucoma patients often experience a profound IOP reduction from cataract surgery alone, both in the chronic [21] and the acute form [22].

We caution against use of phacoemulsification alone for the purpose of IOP reduction as recently advocated [15]. Phacoemulsification on its own does not always lower IOP reliably in glaucoma patients because of a relatively more diseased TM [23] and can cause potentially dangerous IOP spikes during the postoperative course [23,24]. This can be prevented by combining AIT with phacoemulsification [24]. A higher than normal IOP before surgery is a risk factor for IOP spikes even in patients without glaucoma [25]. Eyes with a more decreased outflow facility may be more prone to this but also experience a larger IOP reduction from AIT [26]. The impact of phacoemulsification on IOP in patients with same session TM bypass microstents may be relatively higher compared to ab interno trabeculectomy because of more remaining TM [4] and fewer drainage segments accessed in the former [27].

The raw baseline age differences and higher number of medications between AIT and phaco-AIT groups match the increased incidence of cataracts and glaucoma with age [28]. Following coarsened exact matching, these differences were minimized and allowed a statistically valid comparison with linear regression [29]. It was previously assumed that IOP reduction following AIT is relatively independent of preoperative IOP [7,8] and only limited by episcleral venous pressure and other downstream elements [30]. The linear correlation between pre- and postoperative IOPs seen here suggests that patients with higher baseline IOP may have both a higher TM-mediated outflow resistance and a somewhat higher outflow resistance that is downstream of the TM.

This study had limitations. Instead of a randomized controlled trial, we applied newer statistical methods, *Coarsened Exact Matching* and *Multiple Imputation*, that belong to the class of *Monotonic Imbalance Bounding (MIB)* and make no assumptions about the data generation process [12,31]. This accounts for missing demographic values and avoids losing data thereby increasing the validity and sample size of the study. Additionally, the 12 month follow-up is still a relatively short-term follow-up endpoint for patients with good life expectancy and ongoing ocular disease. The calculator presented here may not apply to the pediatric or juvenile glaucoma population. Interpretations of IOP outcomes presented here have to take into account that phaco-AIT patients in reality have a mixed indication of vision improvement (cataract surgery) with often optional IOP or medication reduction (AIT). As a result, IOPs can be considerably lower if the second of the two average glaucoma medication is not eliminated.

In conclusion, this first glaucoma surgery calculator advises clinicians on IOP after trabectome-mediated ab interno trabeculectomy. Phacoemulsification has only a small additional IOP lowering effect when combined with trabectome surgery. Patients with higher baseline IOP are expected to have a greater IOP reduction.
